# Focal Neurotoxicity Associated With Topical 5-Fluorouracil

**DOI:** 10.7759/cureus.54365

**Published:** 2024-02-17

**Authors:** Ryan M Garcia, Maura Mobilia, Jack B Newcomer, Chase L Wilson

**Affiliations:** 1 Dermatology, University of Kentucky College of Medicine, Lexington, USA; 2 Dermatology, Elkhorn Dermatology, Georgetown, USA

**Keywords:** topical chemotherapy, actinic keratosis, toxic neuropathy, 5 fluorouracil neurotoxicity, 5 fluorouracil

## Abstract

Topical 5-Fluorouracil (5-FU) is an antineoplastic chemotherapy drug used to treat precancerous and cancerous skin growths, such as actinic keratoses (AKs), squamous cell carcinoma in situ, and superficial basal cell carcinoma. The topical agent may rarely cause neurotoxic adverse effects. Multiple cases of systemic 5-FU and capecitabine chemotherapy-induced neuropathies have been reported. However, until now, the topical administration of the drug has not been reported to cause neurotoxicity. We present a case of an 83-year-old male who was prescribed topical 5-FU 5% cream to treat AKs on the left anterior scalp and returned weeks later with the development of focal neurotoxicity in the treatment area. He presented with focal paralysis of the left medial frontalis muscle, with initial loss of sensation followed by intermittent pain and paresthesias, persisting four months after the cessation of therapy. He was referred to a neurologist and received a diagnosis of supraorbital neuralgia. The temporal relationship of symptom onset and the localization of symptoms to the treated area strongly suggests that the medication contributed to the observed neurologic effects. These effects are more likely to be observed in patients with a genetic deficiency of dihydropyrimidine dehydrogenase (DPD), which is responsible for the majority of 5-FU degradation (80%), therefore potentially leading to toxic levels of unmetabolized 5-FU. Providers should be aware of the potentially neurotoxic effects of topical 5-FU in order to properly counsel patients and to consider this as a possible etiology of neurologic deficits in patients using this drug.

## Introduction

Topical 5-fluorouracil (5-FU) is a pyrimidine antimetabolite drug commonly used to treat actinic keratoses (AKs) as field therapy in patients with multiple lesions or as an alternative for patients who do not prefer cryotherapy. While 5-FU is one of the most effective methods for eliminating AKs and preventing their progression to squamous cell carcinoma [1], the drug has also been used systemically as a chemotherapy agent for internal malignancies such as colorectal and breast cancers [2]. 5-FU targets and eliminates rapidly dividing cells in the S-phase by inhibiting thymidylate synthase, leading to the disruption of DNA synthesis, RNA processing, and protein synthesis. Both topical and systemic use of the drug can lead to a wide variety of adverse effects. Systemic toxicities include hematologic, cardiovascular, gastrointestinal, and neurological effects. The most common neurotoxic effect seen with systemic 5-FU is acute cerebellar dysfunction, which causes ataxia, nystagmus, and loss of coordinated movements and speech [3]. Rarely, cases of peripheral neuropathy have been reported in patients receiving either systemic 5-FU or capecitabine, the pro-drug of 5-FU. As systemic absorption is minimal with the topical use of 5-FU, these side effects are far less common with topical therapy. The most common side effect seen with topical 5-FU is localized dermatitis, with burning, pruritus, erythema, and/or hyperpigmentation [4]. From a PubMed review of the literature, topical 5-FU has been reported to exacerbate pre-existing peripheral neuropathy in one patient [4].

We present a case of an 83-year-old male with the onset of complex focal neurotoxicity, impairing both motor and sensory function, affecting the unilateral forehead and scalp following a two-week course of topical 5-FU 5% cream. Based on our review of the literature, we did not observe any additional reported cases of focal neurotoxicity developing due to the use of this agent.

## Case presentation

**Figure 1:**
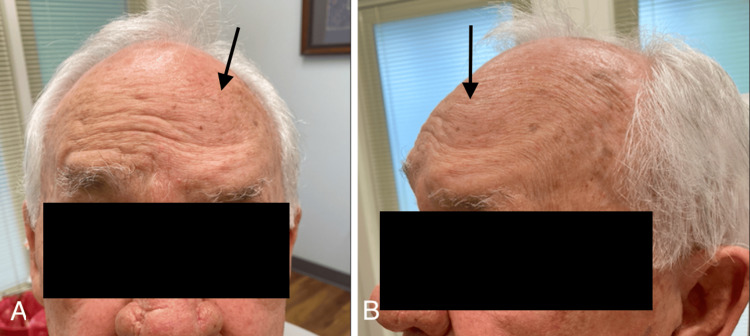
Demonstrates weakness in the left-medial portion of the frontalis muscle when the patient is asked to raise both eyebrows, while the function of both the right frontalis (A) and left-lateral frontalis (B) remains intact.

An 83-year-old male presented to our dermatology clinic for follow-up after treating AKs on the left anterior scalp and left forehead with 5-FU 5% cream twice daily for two weeks. While the medication successfully resolved the AKs, he reported that shortly after completing the two-week treatment period, he developed focal weakness and loss of sensation in the area of the scalp where he had applied the cream. His symptoms began five weeks prior to this visit and persisted without improvement or change in severity. The observed weakness affected a portion of his left frontalis muscle just above the medial portion of the left brow. When asked to lift both eyebrows, he was unable to lift the medial left brow (Figures 1A-1B). The loss of sensation overlaid the same area, roughly the size of the palm of a hand, in the distribution of the left trigeminal V1 nerve branch. He denied experiencing any pain, burning, erythema, or pruritus in this region during or after the treatment period. The patient's past medical history includes cataracts, glaucoma, benign prostatic hyperplasia, and congestive heart failure, with a daily medication regimen that includes atorvastatin, clopidogrel, finasteride, latanoprost, metoprolol, tamsulosin, and timolol. However, he had never experienced a neuropathic condition like this before. Given the temporal association with the development of neuropathy after 5-FU use and the localization of symptoms to the area where the agent was applied, we identified 5-FU as the likely culprit. At this time, the patient was referred to a neurologist to further investigate the etiology of the symptoms.

The patient presented to a neurologist one month later with continued weakness and hypoesthesia in the aforementioned area, and it was also noted that palpation over the left supraorbital notch elicited sharp, reproducible pain. These findings led to a diagnosis of supraorbital neuralgia. The treatment plan included continued observation and reassurance that his symptoms would likely resolve over time. After discussing available treatment options, the patient declined to pursue any other therapeutic measures, as his condition did not cause him significant distress.

The patient returned to the dermatology clinic for a follow-up six weeks after seeing the neurologist and approximately four months since the initial onset of his symptoms. He reported a slight improvement in motor function, despite still being unable to raise his left medial brow. However, he continued to lack all sensation in the affected area with no noticeable improvement.

## Discussion

5-Fluorouracil has three mechanisms of action that ultimately disrupt pyrimidine synthesis and nucleic acid processes. First, 5-FU enters the cell via organic anion transporter 2 (OAT2) and is subsequently converted into an active metabolite known as fluorodeoxyuridine monophosphate (FdUMP). FdUMP binds to and inhibits the enzyme thymidylate synthase (TS). TS normally converts deoxyuridine monophosphate (dUMP) into deoxythymidine monophosphate (dTMP). Inhibition of TS leads not only to a buildup of dUMP in the cell and surrounding tissues but also to the inability of cells to synthesize or repair DNA due to a lack of thymidine [5]. Two other active metabolites of 5-FU include fluorodeoxyuridine triphosphate (FdUTP) and fluorouridine-5-triphosphate (FUTP), which are fluorinated nucleotides that incorporate into DNA and RNA, respectively. Incorporation of these metabolites interferes with DNA replication, RNA translation, and protein synthesis, therefore eliminating rapidly dividing malignant or precancerous cells [6]. However, excessive activation of 5-FU can be cytotoxic to healthy cells, creating the need to limit its availability in the cell. Naturally, the enzyme dihydropyrimidine dehydrogenase (DPD) degrades up to 80% of 5-FU before it becomes active, preventing toxicity in most healthy cells. Patients with a deficiency of this enzyme cannot effectively regulate intracellular active levels of 5-FU and can develop severe neurotoxic side effects [3].

The DPD enzyme is encoded by the DPYD gene, and some individuals have genetic abnormalities that lead to a range in DPD functionality. It is estimated that approximately 5-8% of the total population has some degree of DPD deficiency with varying severity, while roughly 0.2% of Caucasians have a complete loss of enzymatic function. Individuals with these genetic abnormalities are usually asymptomatic and are not aware of their deficiency until they are exposed to capecitabine or 5-FU. It is rare to have symptomatic DPD deficiency, but some patients with more severe degrees of deficiency may experience neurological issues such as delayed cognitive and motor development, leading to intellectual disabilities [7]. Patients with partial loss of DPD function are at an increased risk for adverse effects following the administration of 5-FU, while those with a complete loss of function are at much higher risk and often experience fatal outcomes [8]. Given the dormant nature of DPD deficiency, it is possible that our patient could have been affected by a partial loss-of-function mutation. We considered genetic testing for our patient but ultimately decided against this measure due to its financial burden and the limited benefit it would provide to the patient in this instance.

The focal nature of the neuropathy, in this case, dissuades the idea that 5-FU was systemically absorbed from the skin because adverse effects would be expected elsewhere in the body, not just at the area of application. The temporal nature of our patient’s condition also aligns with other reports of both motor and sensory peripheral neuropathy development following 5-FU and capecitabine treatment. 5-FU and capecitabine-related neuropathies typically begin two to eight weeks after initiating treatment and persist for months or indefinitely after ceasing treatment [9-11]. Neuralgia of the supraorbital nerve, a sensory branch of the ophthalmic division of the trigeminal nerve, could explain the loss of sensation and reproducible pain. However, the partial weakness of the frontalis muscle implies toxicity to a portion of the temporal branch of the facial nerve as well. If the application of 5-FU caused focal neurotoxicity of the supraorbital nerve, it might have also affected the portion of the temporal nerve that innervates the area of weakened muscle.

While it is evident that slow metabolization via DPD leads to cytotoxicity, the complete mechanism of toxicity in neurons by 5-FU is not fully understood. It is important to note that neurotoxicity from 5-FU has been reported in patients without any DPD deficiency, suggesting that multiple mechanisms could lead to similar neurotoxic outcomes [11]. In both animal models and human cases of 5-FU-induced neuropathy, peripheral and central neuron demyelination has been observed [11]. These reports propose another potential mechanism of neuropathy, where Schwann cells and oligodendrocytes are selectively vulnerable to 5-FU toxicity in certain patients. Although it is not known for certain how our patient’s neurological symptoms were triggered, the timing of his symptoms developing shortly after his treatment course with 5-FU, and the localization of symptoms specifically to the area of drug application, strongly suggest a localized neurotoxic reaction from the topical 5-FU. The treatment for 5-FU toxicity involves the immediate discontinuation of the drug accompanied by supportive care [12]. Additionally, uridine triacetate can be used as an antidote for 5-FU poisoning [13].

## Conclusions

Topical 5-FU remains an effective treatment for AKs, often associated with local irritant effects such as erythema, burning, and blistering due to the resulting inflammation from treatment. We present a case of an 83-year-old male who was prescribed 5-FU to treat AKs on the left anterior scalp and experienced focal neurotoxicity in the treatment area. This consisted of partial paralysis of the frontalis muscle accompanied by loss of sensation, persisting four months after the cessation of therapy. Our case illustrates that, although rare, peripheral sensorimotor neurologic issues may occur weeks after using this drug, and recovery from this condition may or may not occur. 5-FU-induced focal neurotoxicity may occur with an increased likelihood in patients with a loss-of-function mutation in the DPYD gene. Providers may consider alternative treatment methods and/or DPYD gene testing before prescribing topical 5-FU to patients who have a personal or family history of adverse outcomes following 5-FU or capecitabine chemotherapy. Additionally, providers should be aware of topical 5-FU as a possible cause of focal neurologic symptoms in order to properly counsel patients and assess localized neurologic deficits.
